# Symptoms of post-traumatic stress disorder in women with history of sexual violence exposure: exploratory analysis of occupational impacts

**DOI:** 10.1192/j.eurpsy.2026.11884

**Published:** 2026-07-31

**Authors:** L. Beltrame, L. M. Kebbe

**Affiliations:** Ribeirão Preto Medical School (FMRP-USP), University of São Paulo, Ribeirão Preto, Brazil

## Abstract

**Introduction:**

Exposure to sexual violence, whether direct or indirect, is strongly associated with the development of post-traumatic stress disorder (PTSD) and significant functional impairments. In Brazilian mental health services, overlapping experiences of sexual violence are frequently identified, either through personal history or indirect exposure within family contexts. Understanding the cumulative effects of these exposures is essential to accurately assess their impact on occupational performance.

**Objectives:**

To analyze the occupational impacts of PTSD symptoms in women exposed to sexual violence, comparing those with a personal history of victimization and those with indirect exposure only.

**Methods:**

This cross-sectional, descriptive, mixed-method study was conducted in an outpatient mental health service. A total of 30 women were assessed using a sociodemographic questionnaire, the PTSD Checklist for DSM-5 (PCL-5) (Osório et al., Rev Bras Psiquiatr 2019; 41:411–418), and semi-structured interviews. Occupational performance domains were classified according to the AOTA framework (AOTA, AJOT 2020; 74:S1–S87). A secondary exploratory analysis compared subgroups based on direct history of sexual violence versus indirect exposure only.

**Results:**

Among the 30 participants, 27 (90%) screened positive for PTSD. Women reporting dual exposure (personal history + indirect exposure) demonstrated significantly higher PCL-5 scores (M=58) compared with those reporting indirect exposure only (M=46), indicating greater symptom severity and broader functional impairments (Image 2). PTSD symptoms in the dual exposure group were also associated with greater disruption in daily routines, role participation, and social interactions. Across the entire sample, the most affected occupational performance domains were rest and sleep, social participation, leisure, work, activities of daily living (ADLs), and care of others (IADLs) (Image 1). These findings suggest that the cumulative effect of repeated exposure may exacerbate symptom severity and compromise adaptive functioning.

**Image 1:**
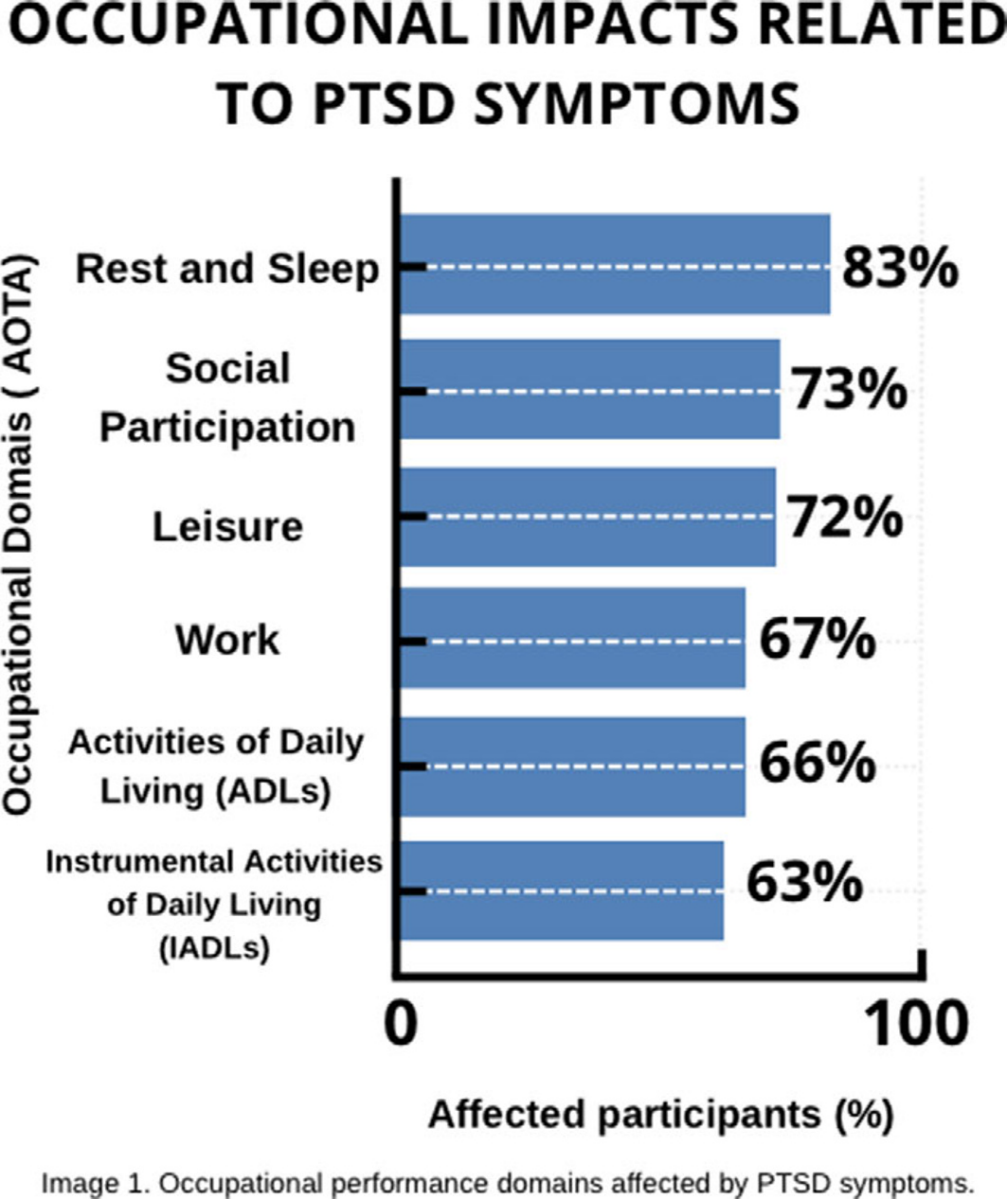

**Image 2:**
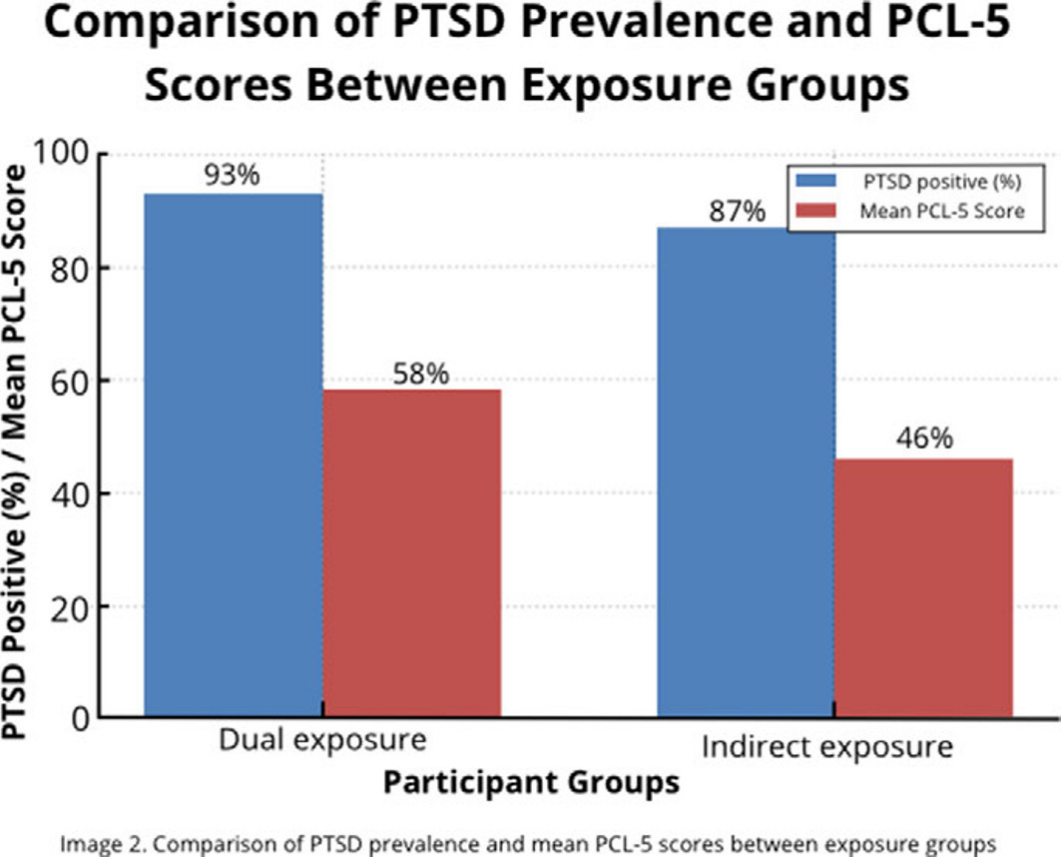

**Conclusions:**

PTSD symptoms were associated with substantial occupational performance disruptions, especially in rest and sleep, social participation, and leisure, reflecting significant limitations in daily functioning. Women with dual exposure exhibited more intense psychological distress and broader occupational impairments than those with indirect exposure only, suggesting a possible cumulative effect of dual exposure.These findings highlight the urgent need for interprofessional, occupation-centered strategies integrating early screening, targeted interventions, and psychosocial rehabilitation to support recovery and foster social reintegration. The results also emphasize the relevance of occupational therapy perspectives in trauma-focused care.

**Disclosure of Interest:**

None Declared

